# Subtractive clustering for spatial resource allocation problems in waste management

**DOI:** 10.1038/s41598-026-45718-4

**Published:** 2026-03-25

**Authors:** Éva Kenyeres, Alex Kummer, János Abonyi

**Affiliations:** HUN-REN-PE Complex Systems Monitoring Research Group, Department of Process Engineering, University of Pannonia, Veszprém, H-8200 Hungary

**Keywords:** Spatial resource allocation, Subtractive clustering, Road network, Waste collection, Resource attractiveness, Engineering, Environmental sciences, Environmental social sciences, Mathematics and computing

## Abstract

A subtractive clustering-based methodology is introduced in this paper, that is applicable to solve spatial resource allocation problems that often arise in waste management. To satisfy spatially distributed demands, resources have to be allocated so that they would have the same spatial distribution. They are often characterized by limited capacity and a catchment area that should also be considered. We propose a flexible subtractive clustering-based methodology that addresses these challenges. The influence of resources and demands on their neighborhood is described by tunable basis functions. Resources are handled as clusters whose centers should be located. The demand arising at a spatial point and its neighborhood is summarized by a potential value that defines the ability to form a new cluster. The main contribution of this paper is a modified subtractive clustering method involving road network-based geodesic distances that is applicable to solve urban and large-scale spatial resource allocation problems as well. A case study about the optimization of textile waste containers in Hungary is introduced to verify the above-mentioned issues. The results show that the proposed method can be used effectively to obtain a resource supply system that is highly consistent with the spatially distributed needs.

## Introduction

In spatial resource allocation problems, the planner aims to allocate limited resources so that they would satisfy spatially distributed demands reasonably and effectively. It means, that resources should be distributed such that they would cover the maximum of demands while the cost is minimal. Since it is a trade-off, the problem type usually determines which of these two goals is considered with greater emphasis. In some cases, uncertainty of certain factors (e.g., costs or demands) is also considered, which makes the problem stochastic. Besides, the space can be considered continuous or discrete, and resource capacity can be constrained in certain cases.

In addition to the above mentioned issues, another main categorization aspect of these problems is the dynamical nature of demands and resources. The type of resource allocation problems when the demand points are spatially and temporally fixed and the location of resources should be selected is called static demand problems generally^1^. Several examples of related real-world problems can be found in the literature. For example, the distribution of thermal energy resources was optimized in two cantons of Switzerland^2^. Otherwise, transportation costs can be minimized by optimal warehouse location^3^. In case of static-resource problems, spatially fixed resources should be distributed among demands. For example, this category includes allocation of natural resources, such as land use or water^4,5^. Lastly, in dynamic resource allocation problems both resources and demands change in time. Cloud computing service allocation or taxi allocation are representative examples for that type^6,7^.

Spatial resource allocation problems often arise during the planning or optimization of waste management systems corresponding to optimizing the location of transfer stations^8,9^ or waste processing facilities (incineration plant, recycling, or landfill sites)^10^. As selective waste collection is increasingly emphasized, container location optimization is also a common problem type^11,12^. These usually can be considered as static-demand problems. In these cases, e.g., the generated waste can be considered as demands that have to be collected or processed in facilities representing resources. General resource allocation aims to distribute the minimum required number of resources among users with uneven (and sometimes even variable) demands. In spatial problems such as waste management facility location tasks, resources and demands are characterized by their attractiveness and mobility, respectively, thus forming geodesic distributions. The task is to determine the optimal locations of resources on the map considering these factors^13^.

State-of-the-art (SOTA) methods use two main approaches to solve spatial resource allocation problems, where the choice mainly depends on the goal to be achieved. On the one hand, it can be handled as an optimization problem where an objective function is explicitly defined, that can be, e.g., the minimization of total travel distance, cost, or time between resources and demands. A solution has also been provided to support considering multiple conflicting goals at the same time^14^. These problems are usually solved by linear programming (LP) or mixed integer linear programming (MILP); those have also been integrated with uncertainty handling strategies to gain robust solutions^15,16^. The main shortcoming of them is that they have high computational costs in case of large problems. On the other hand, clustering-based approaches are also popular, where the goal is to define segments in the investigated geographic region (a city, a region or a country) that contain approximately the same amount of unevenly distributed demands^13^. In this case, the optimal location of cluster centers representing resources are sought for, so that the demands could be assigned to them evenly. For example, resource blocks and power can be allocated by a modified K-means algorithm in ultra-dense networks that has been made applicable to dynamically adjust the number of cluster as well^17^. Otherwise, hierarchical clustering can be used which has been improved by a grid-based technique to make data extraction process more efficient^13^. The utilization of industrial excess heat in district heat network systems has also been investigated using a spatial clustering method^18^. The main drawback of these solutions is that the capacity of resources cannot be considered directly in the basic clustering algorithms. Combined methods can also be found in the literature, which integrates clustering and linear programming methods, where hierarchical clustering is used to decompose a large-scale problem into smaller ones spatially, thus making mathematical programming problem computationally tractable in the subregions and enabling to solve it^2,19^. Moreover, neither LP-based nor clustering-based solutions found in the literature take into consideration the attractiveness of resources and their potentially limited capacity, that could be important factors, e.g., in case of facility location problems related to waste management, making the task more complex than a general covering location problem. This paper aims to propose a solution that involves resource capacity and attractiveness as well, when solving spatial resource allocation problems.

Subtractive clustering (SC) is suggested in this paper as a heuristic optimization algorithm, whose structure is appropriate to model the spatial distribution of resources and demands^20^. In this case, cluster forming ability of potential cluster centers is defined by summarizing the demands arising at the neighborhood of that point, describing them by basis functions. When a new cluster is formed, i.e., a resource is located, its demand satisfying effect in the neighborhood is determined considering its attractiveness and capacity. This method is appropriate not only for the design of resource allocation in an empty area but also for the optimization and complement of existing resources. Only a few works were found that use the SC algorithm for resource allocation; however, none of them was a spatial problem and the role of it was usually only to initialize the number of clusters for another clustering algorithm (e.g., K-means^17^ or fuzzy C-means^21^). This paper uses the SC algorithm directly to solve resource allocation problems, and proposes problem-oriented modifications to make it applicable for spatial problems.

The applicability of the proposed method is illustrated by a case study related to the allocation of textile waste containers in Hungary, but it can be used for any type of facility location problems. Most of the previous works are dealing with city-level resource allocation problems to introduce their solutions; however, regional or country-level examples are rare. Therefore, our suggested method is tested on a country-level problem to show that the proposed method is applicable for not only urban but also large-scale problems.

In case of large-scale spatial resource allocation problems, calculating the distances between demands and sources is also an essential part of the solution. Using absolute distances may be correct at the city level^22,23^. However, the road network strongly affects the accessibility of resources at the regional or country level. Moreover, sometimes travel time is a more representative measure than travel distance regarding this issue, which can also be relevant in the city-level considering traffic jams. This work proposes a methodology that involves information about the road network during the allocation of resources.

The novelty of this paper is a subtractive clustering-based methodology that is applicable to solve spatial resource allocation problems directly. The catchment area of resources and mobility of demands are represented by basis functions, thus resources can be considered as clusters whose center locations are sought for. Thereby, the outlined resource allocation problem is converted to a clustering one, where all clusters can be represented by their capacity, shape of the basis function describing their catchment area, and center location. By the proposed method, the limited capacity of resources and their attractiveness can be considered during resource allocation, overcoming this deficiency of existing methods. Information about the road network is also incorporated into the suggested solution by using geodesic distances on the road network, computed as shortest-path distances. The method is verified by a country-level case study related to the optimization of the textile waste collection system in Hungary to show its applicability for large-scale problems that forms a gap in the literature.

The advantages of the proposed method mentioned above can be quite useful in spatial resource allocation problems related to waste management. Waste has mobility, since people who deliver it to the collection points travel in the municipality or even in its neighborhood on a daily basis. Containers or other waste collection facilities have a limited capacity, and their locations attracts various number of residents. Road network often forms a limit regarding the availability of places considering its structure and the traffic on it as well. The large variety of problems requires a flexible method. Moreover, optimization is often needed not only at the urban, but at a regional or country level as well.

The contributions of the paper addressing the issues mentioned above are the following:

- A method is proposed to handle spatial resource allocation problems in which resources have a catchment area and limited capacity;
- Information about the road network is involved in clustering to calculate realistic distances between demands and resources;
- A flexibly tunable subtractive clustering-based technique is provided whose structure and logic represent that of general practical problems, thus can be easily used for many types of problems by eliminating actually irrelevant factors;
- The applicability of the method is shown for large-scale problems.

The roadmap of the paper is the following: in Section “Methods”, a general spatial resource allocation problem is formalized, the proposed SC-based method is introduced in detail, and some metrics are suggested for the evaluation of results; Section “Results” introduces the large-scale case study of Hungarian textile waste collection system together with the related results of container system optimization, and a sensitivity analysis; in Section “Discussion”, some remarks are given about the advantages, disadvantages and general applicability of the proposed method, and a comparison with alternative state-of-art methods is provided; finally, Section "Conclusion and future directions" summarizes the conclusions based on the results and some ideas about future research directions are also provided.

## Methods

This section introduces how spatial resource allocation problems can be effectively solved using a modified subtractive clustering (SC) technique. After formalizing a general spatial resource allocation problem by defining resources and demands, the basic concept of SC and its problem-oriented modifications are introduced. Some metrics are also provided that are suitable to evaluate the results.

### Formalization of spatial resource allocation problems

In waste management, spatial resource allocation problems often arise. For example, when an existing waste collection system is needed to be optimized, or a new one is planned for the selective collection of a waste component, it is essential to consider how many people can be reached. Nowadays, container allocation problems often appear in selective waste collection, e.g., when extra containers are involved to improve the collection performance and increase their availability. Besides, the definition of waste collection zones is also a similar problem. In these cases, the services provided (e.g., containers, waste management facilities) can be considered as resources, and residents producing waste as demands that have to be satisfied.

In spatial resource allocation problems, demands and resources both can have a spatial distribution. Shopping centers, e.g., usually have a large attractiveness. Thereby, locating a container there can be more popular than one in a less frequented area. On the other hand, residents are mobile, and their ability to reach the service can depend on the location of their home and workplace, their transportation options (e.g., if they have a car) or the availability and flexibility of public transport in the certain area. It is obvious that a facility with a better connection to public transport is preferred over one with similar characteristics otherwise, but more difficult to access. These factors, i.e., mobility of demands and attractiveness of resources should also be considered when the optimal resource allocation plan is sought for.

Demands can be characterized by their spatial location (coordinates summarized in vector $\textbf{x}_i$) and magnitude ($N_i$). If demands (e.g., residents) have the ability to change their position to reach the resources (e.g., containers), their mobility should even be considered. It can be described by a basis function ($f_D(\textbf{x}_i,\textbf{x})$), describing the capability of the demand at $\textbf{x}_i$ to take the distance between $\textbf{x}_i$ and $\textbf{x}$. Thus, each demand ($D_i$) can be formulated as a set of these three factors:

1 $$\begin{aligned} D_i \xrightarrow {} \{\textbf{x}_i,N_i,f_D(\textbf{x}_i,\textbf{x})\} \end{aligned}$$

Thereby, the actual demand arising at a spatial point can be gained if the effects of the needs arising at that point and its neighborhood are also considered. Calculating it for each point of the map, the distribution of the demands can be obtained. The aim of a spatial resource allocation problem is then to settle the resources so that their spatial distribution would be close to that of the demands, while their capacity is sufficient to satisfy the needs.

Resources can be characterized by their spatial location (${\textbf{x}_i}^*$) and capacity (*C*) considering equal for all in this work. Capacity here represents the amount of demands that can be satisfied by a resource. The ability of resources to satisfy demands in the neighborhood is defined by their spatial attractiveness. It can be described by a relevant basis function ($f_R(\textbf{x}_i,\textbf{x})$) representing how much the resource at $\textbf{x}_i$ attracts demand at $\textbf{x}$, being inversely proportional to the cost (e.g., traveling distance or time) of reaching the resource by analogy with Eq. 3. Thereby, resources ($R_i$) can be formulated as:

2 $$\begin{aligned} R_i \xrightarrow {} \{{\textbf{x}_i}^*,C,f_R(\textbf{x}_i,\textbf{x})\} \end{aligned}$$

As introduced, mobility of demands and attractiveness of resources can be modeled by basis function $f_D$ and $f_R$. These are decreasing by the distance of the two objects in the arguments of the function, and thus analogous with the distance-deterrence functions in gravity models^24^. Their shape and parametrization can be determined flexibly depending on the certain demand or resource. For example, they can take an exponentially decreasing characteristic by distance as:

3 $$\begin{aligned} f_X(\textbf{x}_i,\textbf{x}) = e^{-\alpha d(\textbf{x}_i,\textbf{x})} \end{aligned}$$

where $X \in \{D,R\}$ and $d(\textbf{x}_i,\textbf{x})$ refers to the distance between $\textbf{x}_i$ and $\textbf{x}$, that can be defined by any distance metric, e.g., absolute Euclidean ($d(\textbf{x}_i,\textbf{x}) = \Vert \textbf{x}_i-\textbf{x}\Vert$) or squared distance ($d(\textbf{x}_i,\textbf{x}) = \Vert \textbf{x}_i-\textbf{x}\Vert ^2$). However, in large-scale spatial problems, geodesic distances should be used to gain realistic results.

Resources defined according to Eq. 2 can be considered as clusters whose centers correspond to the resource location, and extensions to the catchment area. These are wanted to be placed optimally on the map to satisfy the demands. By considering spatial attractiveness not only as a neighborhood with a certain radius but also taking into account its variability by the distance, the problem forms not a simple covering location problem, and a special mathematical tool is needed to handle it. The concept of subtractive clustering is quite appropriate to solve this type of clustering problem.

### Concept of subtractive clustering under the scope of resource allocation

Subtractive clustering (SC) is a special form of the Mountain Method proposed by Yager and Filev^25^. According to the core idea, potential values are assigned to the potential cluster centers, that represents the ability to form a new cluster. Cluster centers are selected one after another, and the potential values are reduced by the effect of the new cluster center. In case of the Mountain Method, the data space was gridded, and all grid points were considered as potential cluster centers^25^. On the other hand, subtractive clustering defines the *n* data points ($\textbf{x}_i$, where $i=1,...,n$) as potential cluster centers thus avoiding making a grid^20^.

The above idea can be easily transformed to a spatial resource allocation problem. Data samples represent the arising needs in this case, being distributed on the map. Potential values assigned to certain points of the map (representing cluster forming ability) are proportional to the summarized demands from sources located nearby. The formation of a new cluster can be considered as settlement of a new resource. Most of the practical spatial resource allocation problems (e.g., facility location), demand arises from population, i.e., in the municipalities, and resources are supposedly wanted to be settled there, as well. Depending on the problem relating to the urban or regional level, potential cluster centers can be defined by all grid points (Mountain Method) or the coordinates of municipalities being identical with the demand sources (Subtractive Clustering).

Resources are considered as clusters in the proposed SC-based method, whose optimal location should be found. In this work, the demand locations ($\textbf{x}_i$) were considered as their potential location according to the SC method. After defining them (e.g., by the coordinates of municipalities), the initial (cluster forming) potentials of these spatial points should be calculated to be able to choose the location of a new resource. This is defined by summarizing the demands that arise in the neighborhood.

The summarized demands are need to be calculated for all $\textbf{x}_i$ considered as potential values to define new resources in the SC method. Let us denote the summarized initial potential value assigned to $\textbf{x}_i$ by $P^0_i$. It can be obtained by adding up the demands arising in the neighborhood of $\textbf{x}_i$, weighting them by their distance from it. This means that the demands being closer to $\textbf{x}_i$ would get higher weights, and the ones further from it would get smaller or nearly zero weights. The weighting function is conventionally exponential, whose slope can be tuned by the $\alpha$ parameter. The partial potential at $\textbf{x}_i$ generated by the effect of demand at $\textbf{x}_j$ can be formulated as:

4 $$\begin{aligned} P_i (\textbf{x}_j) = N_j f_D(\textbf{x}_i,\textbf{x}_j) = N_j e^{-\alpha d(\textbf{x}_i,\textbf{x}_j)} \end{aligned}$$

where $N_j$ represents the demand emerging at $\textbf{x}_j$, $f_D$ denoted the exponential basis function representing the mobility of demands. Parameter $\alpha$ indirectly defines the width of neighborhood that is considered by setting the slope of the exponential weighting function, i.e., the catchment area in which the demands are assumed to contribute to the potential value of $\textbf{x}_i$. Thus, the definition of $P^0_i$ can be formulated as:

5 $$\begin{aligned} P^0_i = \sum _{j=1}^n P_i (\textbf{x}_j) = \sum _{j=1}^n N_j e^{-\alpha d(\textbf{x}_i,\textbf{x}_j)} \end{aligned}$$

where *n* marks the number of demand points.

After the initial potentials for all $\textbf{x}_i$ were computed, the location of the first resource ($\textbf{x}_1^*$) can be chosen. That is the point with the highest potential value ($P_1^*=\max P^0_i$). The effect of this new resource on the other potential values should be taken into account. When the potential values are updated, they are reduced with the demand absorbed (*C*) by the newly located resource. Of course, this absorption is not point-wise, but it affects the neighborhood of the new resource. Thereby, the potentials are reduced proportionally with the distance from $\textbf{x}_1^*$. This means that the potential values of spatial points near the new resource decrease more than those that are further from it. (The affected neighborhood of the new cluster can be set by the $\beta$ parameter.) Afterwards, the location of the next resource (the one with the maximal updated potential) is chosen, and the potential values are updated again. These steps are repeated until the stopping criterion is satisfied. The potential of $\textbf{x}_i$ after choosing the *k*th cluster center, describing the remaining unsatisfied demand at $\textbf{x}_i$, can be expressed recursively as:

6 $$\begin{aligned} P^k_i = P^{k-1}_i - \min (P_k^*, C)\cdot f_R(\textbf{x}_k^*,\textbf{x}_j) = P^{k-1}_i - \min (P_k^*, C) e^{-\beta d(\textbf{x}_k^*,\textbf{x}_j)} \end{aligned}$$

where the distances are weighted exponentially again as in case of Eq. 5, but the slope is defined by $\beta$ here included in the $f_R$ basis function. $\beta$ can be tuned relative to $\alpha$, e.g., the higher their ratio is, the more the resources are forced to be far from each other. *C* marks the capacity of a resource. (If it is greater than the actual need, i.e., unsatisfied demand ($P_k^*$) at that point, then $P_k^*$ is considered instead.) Consequently, $P^k_i$ does not necessarily decrease to zero, and the same spatial point can be chosen again to settle another resource there, in contrast to the original SC method.

If there are initially settled resources, and the task is to complement them optimally, the initial potentials can be modified by a similar logic as:

7 $$\begin{aligned} \hat{P}_i^0 = \sum _{j=1}^n \bigg ( N_j e^{-\alpha d(\textbf{x}_i,\textbf{x}_j)} - N^R_j C\, e^{-\beta d(\textbf{x}_i,\textbf{x}_j)}\bigg ) \end{aligned}$$

where $N^R_j$ denotes the number of already settled resources at point $\textbf{x}_j$, and $\hat{P}_i^0$ marks the redefined initial potential. In this case, some initial potential values may be negative, referring to the resource surplus at that place, while positive values indicate the deficit. Thereby, the current situation of resource distribution can be easily evaluated as well.

Parameters $\alpha$ and $\beta$ represents the demand mobility and resource attractiveness, respectively. The value of $\alpha$ can be defined, e.g., considering the average mobility of residents per day in case of problems where mobility of demands depends on the mobility of people. Besides, $\beta$ can be defined according to the attractiveness of the different locations, e.g., how far the workers of a city or the costumers of a shop live. Additionally, the proposed method makes it possible to different parameter values for each demand and/or resource, if there is detailed information about it.

The pseudocode of the proposed method is summarized in Algorithm 1. The stopping criterion can be defined by the desired number of clusters, a potential threshold, or the closeness of existing resources can also be involved^20^. The computational complexity for the initialization step is $O(n^2)$; however, after that, one iteration depends in first-order on the number of potential resource locations (*n*) as *O*(*n*). That can be defined for all iterations as $O(K \times n$), which also depends on the number of resources (*K*) needed to be located. In country-level resource allocation problems, resources are usually wanted to be attached to municipalities. These are also the places where demands arises, i.e., potential resource locations and demand locations are the same on the map. In these cases, distances between municipalities can be stored when calculated first time during creating the initial potentials, and they can be used during the iterative part to accelerate the computation. This strategy is especially useful when complex, e.g., road network-based distances are applied. Moreover, the calculation of initial potentials and the potential reduction parts can be parallelized to make the computation faster.

**Algorithm 1:**
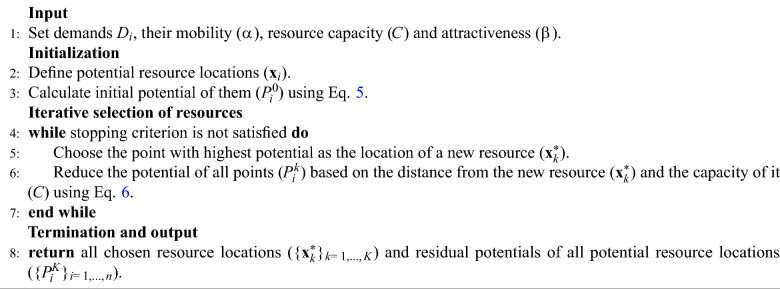
Pseudocode of subtractive clustering algorithm for spatial resource allocation problems.

If ’resource location’ terms are replaced with ’cluster center’ in Algorithm 1, it becomes completely analogous to the original SC algorithm in concept. The technique introduced in this section includes three problem-oriented suggestions that the original SC algorithm does not include:

1. Demands ($N_j$) were introduced as a weighting factor during the computation of initial potentials (see Eq. 4). Originally, the value of a sample point is one; however, we suggest weighting it by the arising demand.
2. Road network-based geodesic distances were recommended to use instead of absolute ones in spatial problems ($d(\textbf{x}_i,\textbf{x}_j)$). These can be calculated by considering the existing road network to determine the shortest distance between $\textbf{x}_i$ and $\textbf{x}_j$.
3. Finite capacity (*C*) of resources (clusters) was considered. Originally, the potential of the new cluster center was reduced to zero and a spatial point could be chosen at most once; however, the proposed method allows to choose a point multiple times if the demand was not completely covered by the settled resource. Furthermore, different capacities of resources can also be considered.

Some metrics for being able to evaluate and validate the resource allocation performance of the proposed method will also be provided in Section Metrics for the evaluation and validation of results.

### Metrics for the evaluation and validation of results

The results obtained by the proposed method should be evaluated to validate the solution. The main aim of a resource allocation problem is to reach a similar resource distribution to the demands while the maximum possible amount of needs is covered by the limited resources. Thereby, our aim was to propose a method that is applicable for settling resources in accordance with the demand distribution while maximizing the coverage. For evaluating the provided solution, some metrics are suggested in this section that clearly characterize the performance of results gained by solving the resource allocation problem, considering different aspects.

Three main aspects can be taken into account to get a full picture:

(A) The *absolute performance of resulting resource distribution* should be determined, also considering the case when there are not enough resources to satisfy all demands.
(B) The *relative performance of the resulted resource distribution* can be gained by comparing the optimal to the actual situation (if some resources have already been settled, that should be complemented or optimized).
(C) The *absolute performance of the actual resource distribution* should be investigated to be able to describe the baseline condition.

These three tasks are different by the underlying mathematical problems as well. In Case (B), two spatial distribution of resources have to be compared that requires relative measures. The questions in Cases (A) and (C) can be answered by defining an absolute metric related to performance, or the corresponding (actual or optimal) resource distributions can be compared to the distribution of demands by some relative metrics. Therefore, we suggest absolute metrics, and even relative ones that are appropriate for comparing distributions of the same or different types.

The following four metrics are suggested here to cover the above-mentioned issues:

1. *Unsatisfied demand ratio* ($\Delta P$): This metric represents the percentage of demands that are not satisfied by the located resources. It corresponds to the potential values remaining after allocation ($P_i$), relative to the initial potential values, and can be calculated by dividing the relevant summarized potentials as:
  8 $$\begin{aligned} \Delta P=\frac{\sum _{i=1}^n P_i}{\sum _{i=1}^n P_i^0} \end{aligned}$$
  It has to be noted that this metric is strongly model-dependent. Since it is calculated from the potential values, its value is influenced by the basis functions chosen to describe demand mobility and resource attractiveness, as well as its parameters and the distance metric used. However, it characterizes well what percent of demands are satisfied, if the defined model is assumed a reasonably accurate approximation.
2. *Surplus* ($S(\textbf{x}_i)$) *and deficit* ($D(\textbf{x}_i)$): These are the simplest metrics when comparing two discrete distributions of the same type, e.g., two different resource allocation plan, or the optimal and the actual one. Subtracting the number (or summarized capacity) of resources settled at $\textbf{x}_i$ according to the optimal plan ($R_i^*$) from that of the actually settled resources ($R_i$), the surplus or deficit at $\textbf{x}_i$ can be gained, depending on the result is positive or negative, respectively.
  9 $$\begin{aligned} |R_i-R_i^*| = {\left\{ \begin{array}{ll} S(\textbf{x}_i), & \text {if } R_i-R_i^* \ge 0\\ D(\textbf{x}_i), & \text {if } R_i-R_i^* \le 0 \end{array}\right. } \end{aligned}$$
  If the computed difference is zero, than the number of resources is optimal at $\textbf{x}_i$.
3. *Jaccard-index* ($J(\cdot ,\cdot )$): This metric is appropriate to compare two discrete distributions of the same type, e.g., that of the resources, with the same number of elements. Its basic idea is to form a ratio by dividing the number of common elements at a point in the two distributions or sets (intersection) by maximum of elements (union)^26^.
  10 $$\begin{aligned} J(\text {actual},\text {optimal}) = \frac{\sum _{i=1}^n \min (R_i,R_i^*)}{\sum _{i=1}^n \max (R_i,R_i^*)} \end{aligned}$$
  The value of it tends to one if the two distributions are similar to each other, since in that case the two distributions have approximately the same elements at each $\textbf{x}_i$ spatial location ($R_i \approx R_i^*$), i.e., their minimum and maximum are close to each other for all *i*; otherwise, *J* takes zero if the two distributions are completely different, which means their elements appear at different spatial locations and the number of common elements tends to zero at each $\textbf{x}_i$. The drawback of this metric is that although it gives information about the similarity of resources among demands by a quite simple and rapid calculation, it ignores spatial similarity.
4. *Wasserstein distance* ($\textit{WD}$): A metric for comparing two distributions that eliminates weaknesses of the Jaccard-index. In this case, spatial conditions are also considered, i.e., how much it costs to transform one of the distributions to the other. Wasserstein distance is a generalization of Earth Mover’s Distance (EMD) introduced by a Russian mathematician in 1969 to solve optimal transport problem on probability spaces^27^. While EMD works on discrete spaces, Wasserstein distance is applied for continuous spaces, making it possible to consider the cost factor at higher orders^28^. If the demand and resource distributions are transformed to probability distributions, they can be compared appropriately with this metric, e.g., in Case (A) or (C) considering spatial similarities besides magnitude as well. The transformation is done by normalization in this paper, that means that the magnitude of demands (or resources) at each municipalities are divided by the total value, thus discrete probability distributions are gained on the map. Euclidean distance is used here as as a ground metric. The value of $\textit{WD}$ tends to zero if the two distributions are the same. However, it does not have a maximum value as it represents the mass transport needed between the distributions to transform one into the another, and it cannot be normalized in a representative way, which is a main drawback of it. Therefore, it is informative together with other metrics, or when different solutions are wanted to be compared.

**Fig. 1:**
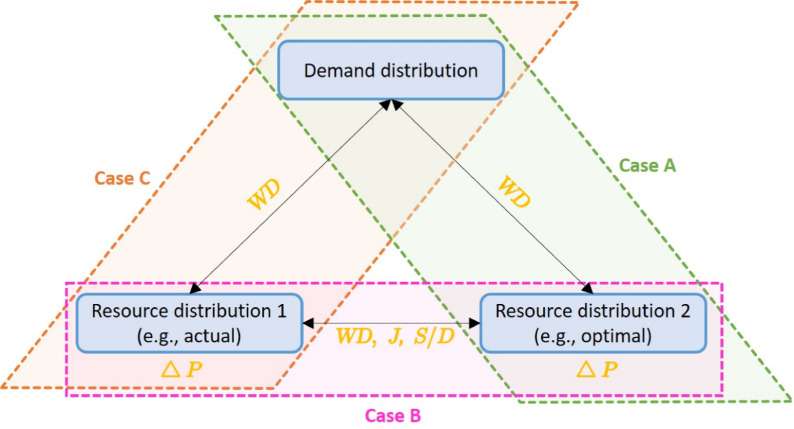
Concept map of the applicability of suggested metrics for describing connections between demand distribution, actual resource distribution and optimal plan. *S*/*D* denotes the surplus and deficit metrics together. Cases (A)-(C) refer to the three aspects of evaluation mentioned above.

The metrics above can be categorized to absolute and relative ones, and they can be used for measuring different factors according to their advantages and limitations. $\Delta P$ is the one that can characterize the resulted resource distributions standalone as an absolute metric. It can also be used to evaluate the actual resource distribution. $S(\textbf{x}_i)$ and $D(\textbf{x}_i)$, such as the Jaccard-index are appropriate for comparing two resource distributions, e.g., two resource allocation plans or the optimal plan with the actual one, being relative metrics. Surplus and deficit can be interpreted graphically as they are assigned to spatial points, while Jaccard-index expresses the dissimilarity numerically. Wasserstein distance is excellent for measuring the distance of resource distributions from the demand distribution and thus comparing them. The improvement of solutions can be detected quite well with this measure. The metrics can be used for characterizing links between demand, actual and optimal resource distributions are summarized in Fig. 1.

The three aspects defined at the beginning of the section (Cases (A)-(C)) are also presented in Fig. 1. The related metrics for all aspects are marked; thereby, the actual and the resulted (optimal) resource distributions can be characterized by $\textit{WD}$ and $\Delta P$ regarding their demand satisfaction, while they can be compared by $\textit{WD}$, the Jaccard-index (*J*) and the surplus/deficit (*S*/*D*) metrics. In the Results section, these metrics were used to validate the proposed method and evaluate the resource allocation results of the introduced practical case study.

## Results

The applicability and performance of the proposed subtractive clustering-based method is demonstrated by solving the optimization problem of the textile waste container system in Hungary. It can be considered as a standard facility location problem: demands represent the amount of generated textile waste here, and the waste collection capacity of containers forms resources. Textile waste is transported to containers by people; therefore, its mobility depends on the mobility of residents. On the other hand, the location of resources has attractiveness, that decreases by distance. Thereby, the basis function representation of demand mobility and catchment area of resources is useful to handle them correctly.

This work deals with the problem on the country level, i.e., optimal distribution of containers among municipalities. The spatial distribution of settlements in Hungary includes a capital, its agglomeration and sparse regions as well, so it is a representative geographical structure and makes it possible to investigate the applicability of the method generally. The textile waste generated is assigned to municipalities as demands, and the aim is to determine in which municipalities and how many containers should be placed. This covers the task of the optimization of already settled containers.

The subtractive clustering concept is applicable to solve resource allocation problems with the conditions mentioned above, as was introduced in the Methods section. Its applicability and performance will also be verified in this section by a practical case study.

### Problem definition based on the actual container system

Transformation to circular economy aims to increase the ratio of recycling over landfill^29^. This requires selective waste fractions; therefore, the selective collection of textile waste is a current topic today. European Union also forces its members to follow this direction by regulations expect to collect their textile waste selectively from the beginning of 2025^30^. Additionally, there are technological reasons besides environmental ones as well; textile waste can cause breakdowns in waste separation systems by rolling up to the rotating parts of machines.

As textile waste cannot be separated effectively from municipal solid waste (MSW) in waste separators, it is often collected selectively by a container system. This means, that containers are located at certain points in certain municipalities, and residents should take their waste there. Of course, their willingness to collect their textile waste selectively and not to put it to the MSW strongly depends on the distance and availability of nearest containers.

**Fig. 2:**
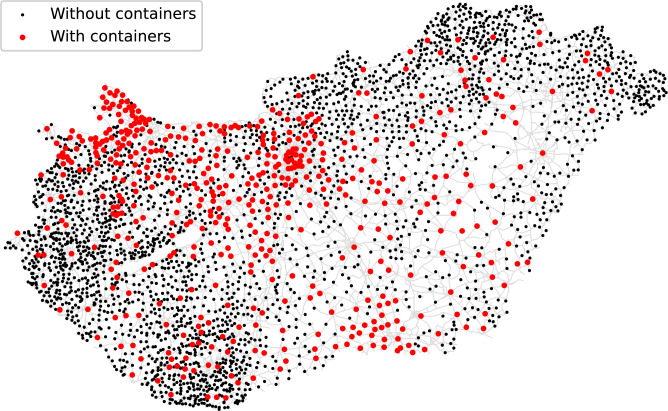
Municipalities of Hungary together with the road network. Those where there is at least one container are marked by red; the others are shown in black. (The map was generated by the authors in Python using the OSMnx package (v2.1.0, OSMnx) from OpenStreetMap road network data. Geographic data are provided by $\copyright$ OpenStreetMap contributors.).

Unfortunately, the container system is quite incomplete in Hungary. The case study was carried out at the beginning of 2025, when there were 2,453 containers settled, distributed among 503 municipalities as seen in Fig. 2. (Budapest, the capital of Hungary, is considered by districts here.) However, since there are 3,155 municipalities (3,177, if Budapest is considered by districts) in total in Hungary, most of them did not have any containers.

**Fig. 3:**
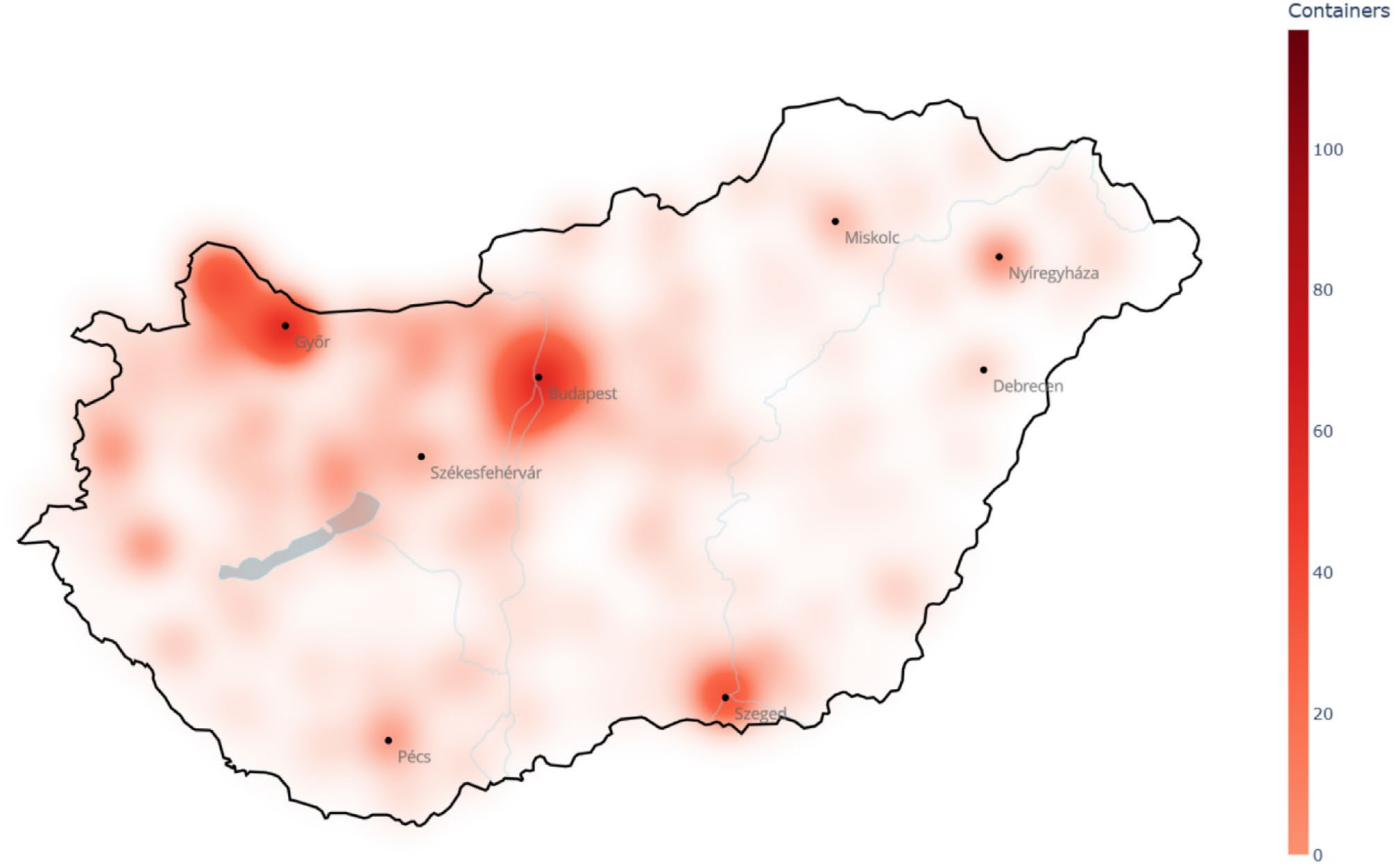
Smoothed spatial intensity map of actual container distribution in Hungary. (The map was generated by the authors in Python using the Plotly Express package (v6.6.0, Plotly). Underlying geographic data are provided by $\copyright$ OpenStreetMap contributors.).

**Fig. 4:**
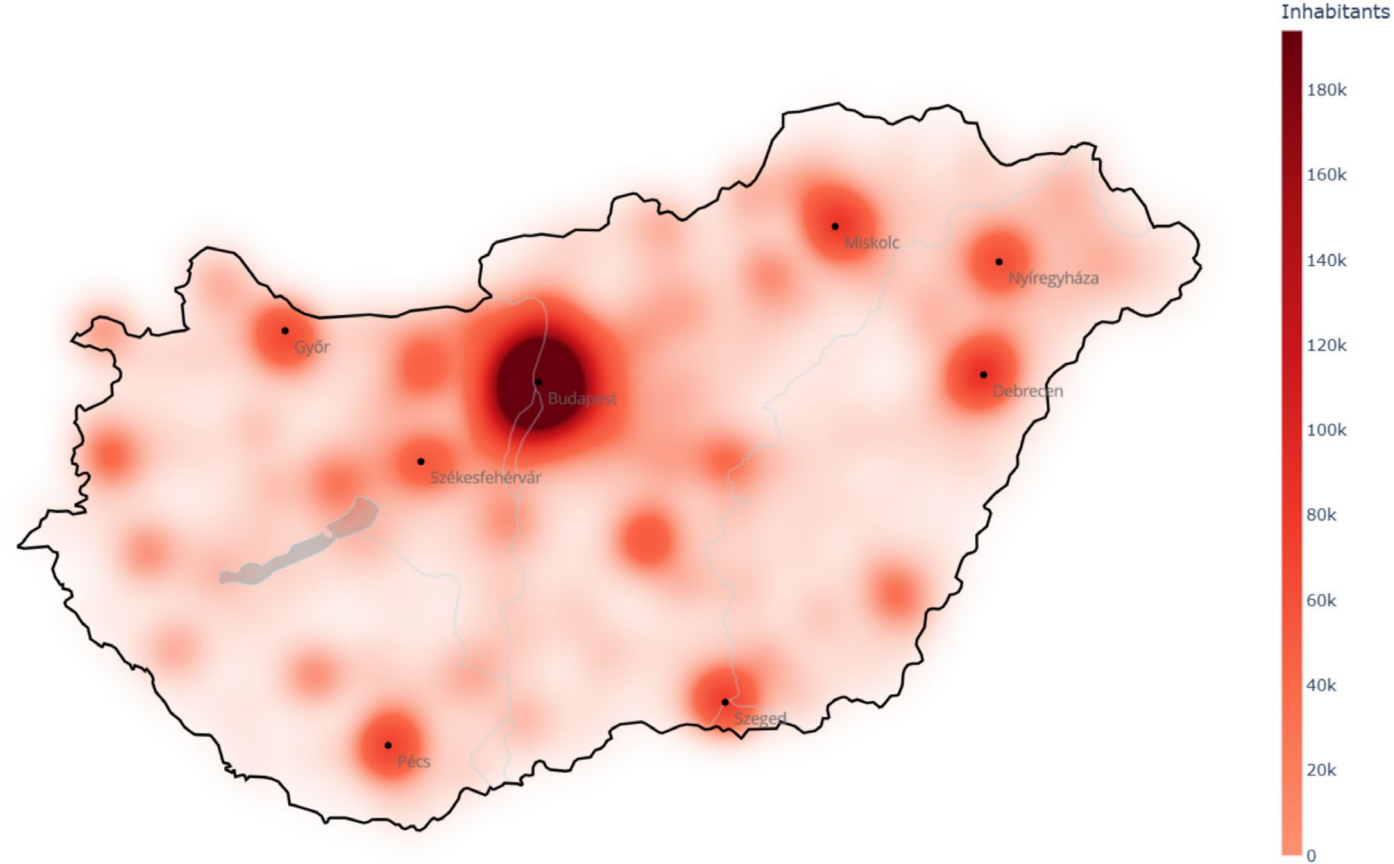
Smoothed spatial intensity map of population distribution in Hungary. (The map was generated by the authors in Python using the Plotly Express package (v6.6.0, Plotly). Underlying geographic data are provided by $\copyright$ OpenStreetMap contributors.).

The generated amount of textile waste is assumed to be proportional to the population in this work. Comparing the actual container distribution (Fig. 3) to the population distribution (Fig. 4) in Hungary, it is clear that they are quite different. For instance, the northwest part of the country is unreasonably overrepresented by containers compared to the other regions, while the northeast part is highly underrepresented. The $\textit{WD}$ between the demand and actual container distribution is 0.55 that also verifies the non-optimality of the current situation, i.e., refers to uneven container distribution. This gives the baseline in the case study.

Regarding the issues mentioned above, there are two main goals for improving the actual textile container system. Availability of containers should be maximized by distributing them optimally (according to the population distribution), so that they reach as many people as possible. Besides, their capacity should be extended to cover all demands. As the container distribution at the country level is not optimal, the first task is to allocate them optimally among municipalities, that is in the focus of this paper.

### Optimization of the actual container system

As was shown in Figs. 3 and 4, the actual container distribution strongly differs from the population distribution (assumed to be proportional to the amount of generated waste). Therefore, the first goal was to optimize the position of the current 2,453 containers using the proposed technique, and investigate how much the obtained solution differs from the actual situation. The stopping criterion of the algorithm was the number of containers wanted to be settled in this case.

The subtractive clustering based strategy for resource allocation proposed in the Methods section is used to solve the container allocation tasks introduced. In this case study, populations of municipalities are used as weights ($N_j$) when calculating initial potential values by Eq. 5. Accordingly, the container capacity is defined here in person per container dimension, defining the number of people whose demands can be covered by a container. The capacity of containers (*C*) is assumed to be equal. The parameters $\alpha$ and $\beta$ that define the slope of exponential basis functions representing demand mobility and resource attractiveness are set equal in this case study ($\alpha =\beta =8.06 \cdot 10^{-5}$), so that the mean of the function would correspond to the average mobility per person per day (that is 12, 400 meter). Of course, different values can also be used conveniently.

Distances between location of demands and resources ($d(\textbf{x}_i,\textbf{x}_j)$), i.e., containers and residents in the neighboring municipalities, are determined as the shortest distance (length-weighted) on the existing road network. For this purpose, OpenStreetMap data was used^31^. The main roads were considered that are accessible by car, including motorways, trunks, primary, secondary and tertiary roads, assuming that they describe the existing connections between municipalities satisfactorily (data extraction date: 4 February 2025). The OSMnx and NetworkX Python packages were used to build the graph and compute origin-destination distance. More details can be found in the shared code at https://github.com/kenyevica/Subtractive-clustering_Textile-waste-containers.

Container capacity (*C*) can be user-defined if there is an approximate estimate about it (e.g., based on emptying intervals of containers, their filling level, and generated waste per person per time). Otherwise, in the absence of empirical data, *C* can be used as a normative control in the algorithm ($C=3981$). If the task is to settle a fixed limited number of containers, a flexible value can be used by dividing the whole population wanted to be covered with the number of containers. This definition is also more preferable when there are some municipalities with very high population (demand) compared to the others and there are not enough containers to satisfy all demands, however, they would like to be distributed evenly in the country. In case of a fixed constant value of *C*, the municipalities with extremely high demands (e.g., the capital or big cities) would get the majority of containers, as their potential would not be reduced rapidly during the search due to the limited total capacity of resources. The definition of *C* assuming all the demands can be covered latently ensures that municipalities with smaller population would also get containers, not only the extremely high ones, thus their distribution would become more even, and corresponding to the demand distribution. Thereby, it is a more advantageous strategy when limited number of resources with insufficient total capacity should be placed, that is the situation in this case study. Thus, *C* acts as a normative control here, rather than a physical throughput estimate.

The parameter values used for the case study in this section are summarized in Table 1.

**Table 1:** Parameters for the optimization of actual container system.

Parameter	Value
*n* [-]	3177
*K* [-]	2453
$\alpha$ [m$^{-1}$]	$8.06 \cdot 10^{-5}$
$\beta$ [m$^{-1}$]	$8.06 \cdot 10^{-5}$
*C* [person/container]	3981

**Fig. 5:**
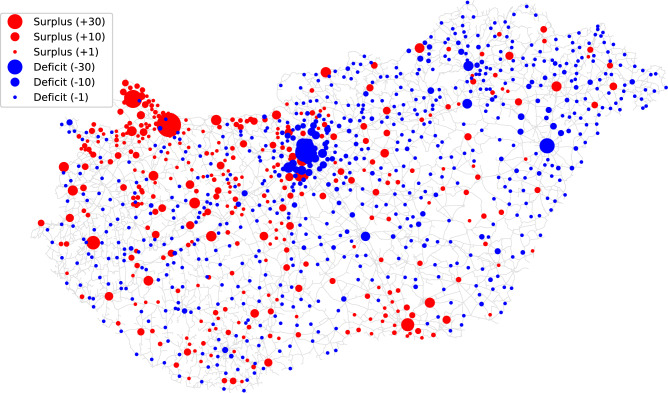
Surpluses (red) and deficits (blue) of the actual container system compared to the optimal one with the same number of containers gained by the proposed method when *C* is used as a normative control being the ratio of total population and limited number of containers. The size of the circles are proportional to the magnitude of surplus or deficit. The road network is illustrated by black lines. (The map was generated by the authors in Python using the OSMnx package (v2.1.0, OSMnx) from OpenStreetMap road network data. Geographic data are provided by $\copyright$ OpenStreetMap contributors.).

The resulted container system was compared with the actual one. In the solution obtained, the 2,453 containers are distributed between 999 municipalities. From these, there are 349 that already had container(s) in real, 650 that did not have any but should according to the optimal system. The municipalities with surplus or deficit were identified, and illustrated in Fig. 5, and Table 2 summarizes the results numerically.

**Table 2:** Numerical evaluation of the resulting optimal container system.

Metric	Value
Maximum deficit [-]	55
Maximum surplus [-]	85
Ratio of municipalities with non-zero deviation [%]	33.84
$J(\text {actual},\text {optimal})$ [-]	0.50
$\textit{WD}(\text {actual},\text {optimal})$ [-]	0.57

It can be seen in Fig. 5 that there is a surplus in the northwest and southeast part of the country, and there is a deficit in the northeast region regarding the optimal container distribution, as was also expected according to Fig. 3 and 4 previously. The maximum deficit is 55 and the maximum surplus is 85 (see Table 2). The Jaccard similarity of the two container location lists is 0.50, while their Wasserstein distance is 0.57. These results also confirm that the actual system is quite far from optimal.

The performance of the proposed method in allocating resources corresponding to the demand distribution can be verified by comparing the population distribution with the resulted container distribution. $\textit{WD}$ is an appropriate metric for this aim, which gives 0.02 in this case, showing that the containers were placed according to our aims by the developed subtractive clustering-based method. Since it is much lower than the baseline situation, it can be concluded that the method is effective to optimize existing container systems. Thereby, although in most of the municipalities (appr. $66\%$, see Table 2), the number of containers is already in accordance with the optimal (mainly in villages where there are no containers), their rearrangement in the remaining ones could bring benefits in getting closer to the demand distribution.

**Fig. 6:**
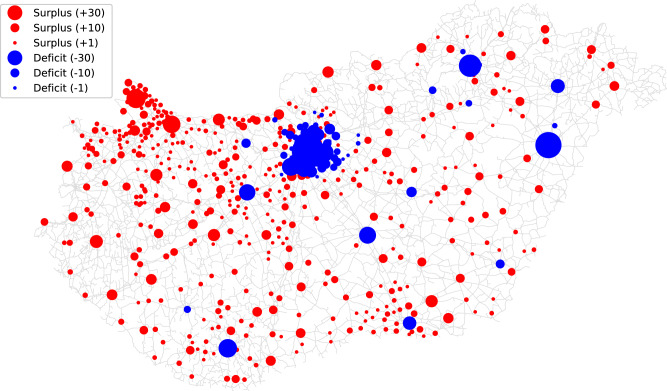
Surpluses (red) and deficits (blue) of the actual container system compared to the optimal one with the same number of containers when a fixed value of *C* is used. In this example, $C=1500$ person per container was used instead of $C=3981$ person per container corresponding to the total coverage of demands. The size of the circles are proportional to the magnitude of surplus or deficit. The road network is illustrated by black lines. (The map was generated by the authors in Python using the OSMnx package (v2.1.0, ) from OpenStreetMap road network data. Geographic data are provided by $\copyright$ OpenStreetMap contributors.).

As a note to verify the above mentioned issues about defining container capacity parameter (*C*) correctly: if a fixed value (smaller than the one got when all the potentials are covered) is used (e.g., $C=1500$ person per container), then the results presented in Fig. 6 are obtained. These results show that in case of a relatively small *C*, the containers in fact concentrate in large municipalities, that is quite disadvantageous regarding spatial coverage ratio of the country. Therefore, if it is also an aim that the spatial coverage of insufficient resource capacity would be the best besides satisfying the demands, *C* is recommended to be determined assuming full demand coverage, i.e., as the ratio of total population and number of containers. Thus, the even distribution of containers can be ensured, corresponding to the population (demand) distribution.

On the other hand, metric $\Delta P$ has relevance only if *C* has a fixed value. (If *C* is used as a normative control, it represents a latent container capacity that does not have a physical meaning.) Some investigations were made on this using $C=1500$. The baseline in this case is $\Delta P = 64\%$ belonging to the actual container distribution. $\Delta P=52\%$ was obtained for the optimal distribution of containers. This means that the unsatisfied demand ratio could be reduced by simply the rearrangement of the existing containers. If the actual container number is increased, then $\Delta P$ decreases more significantly, e.g., $\Delta P=5\%$ if $K=6000$.

### Sensitivity analysis on mobility

The $\alpha$ parameter representing the catchment area of resources (defined as the reciprocal of average mobility) can have a great influence on the results. The smaller its value (the higher the mobility), the resources have a demand-satisfying effect on a wider neighborhood. On the other hand, demands from further also affect the potential value of locations.

**Fig. 7:**
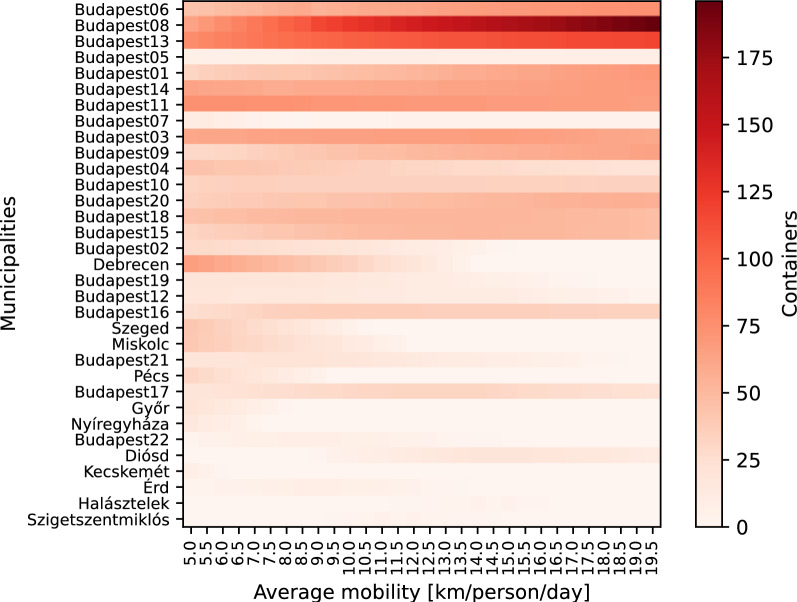
Distribution of containers among the municipalities, under different average mobility affecting $\alpha$ parameter.

To investigate these effects, a sensitivity analysis was performed on $\alpha$. It was changed corresponding to the range of 5,000 to 19,500 meters per person per day average mobility. Parameter $\beta$ is still considered equal to $\alpha$. In this case study, changes in the optimal distribution of the first 1000 containers (without initially settled containers) were investigated ($K=1000$). The other parameters were taken from Table 1. In Fig. 7, the municipalities where a container was settled under either value of $\alpha$ are illustrated.

Figure 7 shows the distribution of containers between municipalities (vertically) for certain mobility values. As mobility grows, catchment area of containers becomes wider. Thereby, containers are rather allocated to the districts for Budapest, while at small mobility values other big cities (e.g., Debrecen, Miskolc, Pécs) around the country also get some. The reason for this is that the districts of Budapest are close to each other. They are supposedly not close enough to be considered in a 5,000 m catchment area of each other, but near enough to be included in the 19,500 m wide neighborhood. Consequently, the demands of neighboring districts are considered more and more as the catchment area grows; thus the potential values of Budapest districts become higher than those of cities in the countryside that do not have any high-population municipalities in the neighborhood.

The issues mentioned above highlighted that the choice of the $\alpha$ parameter, e.g., corresponding to mobility, has a great impact on the results. Therefore, its value should be chosen carefully and realistically to obtain correct results.

## Discussion

In this section, the main advantages and limitations of the proposed method are discussed, and some guidelines are given about the general applicability of the method. Moreover, the proposed SC-based method is compared with other state-of-art methods, and the theoretical claims are supported by some numerical results.

### Advantages and limitations

The results verify that the improved version of subtractive clustering concept is an appropriate tool to solve spatial resource allocation problems. Demand mobility and resource attractiveness could be effectively incorporated by representing them by basis functions, thus they could be considered during the search. Thereby, resources could be allocated corresponding to the latent demand distribution that was verified by the suggested metrics for performance evaluation.

The proposed method is quite advantageous, as it can be easily tuned and applied for any type of spatial resource allocation problem with some problem-specific modifications. For example, it can enhance the planning of a selective waste collection system or the allocation of waste separators or transfer stations in waste management. Resources with limited capacity can be handled, such their attractiveness and mobility of demands. Information about the road network can be involved that ensures realistic results in large-scale problems, as well. Using the suggested metrics, the results can be evaluated effectively, and different resource plans (e.g., actual and optimal) can be compared. These contributions make the method more flexible and enhance to give realistic solutions for practical problems.

The main limitation of the method is that it requires a lot of data, e.g., about demands, attractiveness/mobility, road network. However, as it is quite flexible, there are simple alternatives in most cases to give approximate results, as was will be introduced in Section 4.2. On the other hand, the computation of the Wasserstein distance ($\textit{WD}$) for performance evaluation can be time consuming in case of a lot of municipalities. Then, the graphical comparison or other metrics (e.g., unsatisfied demand ratio ($\Delta P$)) can help.

### Applicability of the method

Although this work introduced a country-level case study, the proposed method for spatial resource allocation can be used for urban planning problems, as well. Analogously, the potential resource locations should be defined first, those could be e.g., the road crossings or some shopping centers, depending on the type of “resources” wanted to be placed. The demands usually also arise in proportion to population in this case. The resources can be containers, waste collection or processing facilities in case of waste management problems; however, it can be applied in other fields as well. Thereby, it can also support the 15-minute city concept^32^.

The proposed method can also be modified according to the particular problem and the available database. For example, $\alpha$ can be determined from the attractiveness of spatial points instead of mobility. If wanted, different values can be assigned to each point. Thereby, e.g., the wider neighborhood of municipalities with a larger catchment area (smaller $\alpha$) would be considered in a country-level problem.

Demands ($N_j$) and resource capacity (*C*) accordingly can be given in a different unit. For example, the type of demand can be used, or another factor with which it is proportional. In the presented container location case study, e.g., the generated waste amount could be used, if there is direct information about it. It can be relevant if in different regions of the country the waste generated per resident is not the same. Of course, the unit of *C* should also agree with that of the demands (e.g., it would be waste amount per container in the above example).

Road network analysis to calculate distances can be useful in both urban or regional problems. Instead of travel distances, travel times can also be used if there is available data about it. Thus, motorways could be considered in the country, e.g., while in case of an urban design problem, information about traffic jams could be involved. Thereby, a dynamical analysis during a workday can also be performed.

Using these extensions, the proposed method is applicable for a wide range of spatial resource allocation problems. It is universal regarding the resource type and scale of the problem as was introduced, and its parameters can be flexibly tuned based on the specific problem.

### Comparison with alternative methods

As was discussed in the Introduction section, some methods based on different concepts have already existed for spatial resource allocation problems. The two main groups of solutions are (mixed-integer) linear programming and K-means clustering. These are summarized in Table 3, compared to the proposed subtractive clustering based method.

**Table 3:** Comparison of existing techniques for spatial resource allocation problems and the proposed subtractive clustering based method.

Method	Advantages	Disadvantages	Initial data needed	Mobility/Attractiveness
Linear Programming (LP, MILP)	– well-established algorithms – discrete decisions are allowed – resource capacities and demands can be considered – can optimize the number of resources	– complicated problem description – all constraints needed in linear form description – high computational demand	– demand locations and values, resource capacities – objective function and constraints defined explicitly	– cannot be considered directly, only by crisp constraints
K-means Clustering	– computationally effective – defines explicitly which resource satisfies a certain demand	– number of resources (clusters) is not optimized inherently – considering resource capacity is complicated	– demand locations and values – number of resources	– cannot be considered directly in the basic algorithm, crisp constraints can be added in a complicated way
Subtractive Clustering	– resource capacities and demands can be flexibly set – the variability of attractiveness and mobility by distance can be considered, and they can be various for the items as well	– potential resource locations should be defined	– demand locations and values, resource capacities – attractiveness of resources and mobility of demands	– defined by tunable basis functions

Linear programming techniques describe the problem by linear objective functions and constraints. Although their solution is well-established, the explicit mathematical formalization of problems (especially in large scale cases) can be a difficult task. The two main subtype of mixed integer linear programming (MILP) problems used to describe resource allocation tasks are locations set covering and p-median. Location set covering technique aims to locate resources so that each demand would be within a certain distance one of them, thus it ensures the availability of resources for all the demands. Resource capacities and size of demands are not taking into account in this solution. Capacitated p-median problems eliminate this shortcoming. They aims to minimize the the sum of distances between demands and resources. The distances can be weighted by the demands, and resource capacities can be considered as constraints. The main shortcoming of MILP methods is that it can model the mobility of demands only by a crisp value: the resources closer than this value can be reached surely, while the ones outside this spatial limit cannot be reached at all. This is not so realistic in a world when mobility of people can be very different depending on their economical status, the place they are living, or road network there as well. Moreover, their willingness to travel strongly depends on distance. Therefore, a trajectory decreasing moderately by distance (that is used in the proposed SC algorithm) seems more preferable to model mobility and also attractiveness.

K-means clustering is also a commonly used technique for facility location problems. It aims to assign demand points to optimally located resources (cluster centers). In this case, the limited capacity of resources cannot be considered, and the required number of resources is not optimized inherently. At maximum one facility can be assigned to each spatial point, that is not preferable if the resources have fix capacities, e.g., in case of containers. Moreover, the demand mobility and resource attractiveness cannot be considered directly using the basic algorithm, only by some complicated extensions those would increase modeling and computational costs significantly.

These deficiencies are eliminated by the proposed subtractive clustering based method, providing a transparent, adaptable heuristic that performs capacity-aware iterative selection effectively. The main contribution of it is the possibility to easily consider mobility and attractiveness as a function of distance. It also allows to use different values for each item, and capacities can also be considered. Although potential resource locations have to be defined initially that may form some limitations, it can be done by making a grid on the map as well. The initial computational effort may be somewhat larger than in the other techniques (as potential values for all the potential resource locations have to be computed); however, the optimization is more straightforward and computationally effective. It is also flexible regarding number of resources, and the order of them is prioritized.

To compare the performance of the proposed method with the other alternatives, we performed numerical experiments. The aim was the same in all the cases: 2453 containers should be placed optimally. Municipalities were considered as potential resource locations in each case. On the one hand, this problem has been defined as a multi-supply capacitated p-median problem as a MILP problem^33^. Thereby, it has been considered that demands in a municipality can be considered by multiple resource locations. On the other hand, the described container location problem has been defined as a K-medoid problem as well. In this case, potential resource locations could be defined discretely.

The results of the comparison with alternative methods can be seen in Table 4. Optimal solutions gained by the proposed subtractive clustering method, the K-medoid method and MILP are compared. According to $\textit{WD}$ between the obtained container plan and the demand distribution, the K-medoid method (with $\textit{WD}=0.60$) gives significantly worse results than the SC, and it is even further from the demand distribution than the actual container distribution. The solution by MILP ($\textit{WD}=0.01$) shows similar performance as the one belonging to the SC algorithm ($\textit{WD}=0.02$), however, the computational time is ten times higher in case of the MILP.

**Table 4:** Comparison of the optimal container plans gained by the proposed SC algorithm and alternative methods.

Container distribution	$\textit{WD}$ from demands [-]	Computational time [s]
actual	0.55	-
SC	0.02	36
K-medoid	0.60	2.4
MILP	0.01	357

Considering the issues mentioned above, the proposed subtractive clustering technique is applicable to allocate resources corresponding to the demand distribution. It overperforms the solution by K-medoid method, and more faster than the MILP solver. Moreover, it allows to consider and flexibly handle resource attractiveness and demand mobility.

## Conclusion and future directions

This work introduces a flexible methodology based on subtractive clustering that is applicable to solve spatial resource allocation problems. The technique is suitable for both urban and large-scale problems. Optimal resource allocation means that limited resources need to be settled so that their distribution would follow that of the demands (while covering the maximum of demands). In spatial problems, the distance between demands and resources is also a crucial factor. Demands can have mobility (e.g., related to mobility of residents), and resources can be characterized by their attractiveness, whose intensity depends on the distance from their location. These can be represented by basis functions; therefore, a special method is needed during the optimization, that is able to incorporate these.

It was shown in this work, that the subtractive clustering concept with some modifications is an appropriate tool to solve the problem outlined above. The main idea of the proposed method is to assign potential values to the locations where resources can be settled, considering the demands in their neighborhood as well, using the basis functions representing mobility. Potential values represent the chance of settling a resource at the spatial point in our method. The location of the new resource is always chosen related to the maximal potential value, and the others are updated based on its demand-satisfying effect considering the basis function representing its attractiveness. The main improvements over existing methods are the incorporation of demand mobility, resource attractiveness and limited capacity into the solution. In addition, road network-based distances were involved to ensure realistic results. Some metrics for performance evaluation were also suggested, revealing their limitations as well.

An illustrative example of the optimization of the textile container system in Hungary was shown to verify the effectiveness of the method. The results were evaluated by the suggested metrics. It has been seen that the method is applicable for rearranging and thus optimizing an existing resource system, based on spatial distribution of demands. Data about road networks were involved, to ensure realistic results and support practical problem solving. Sensitivity analysis on the mobility parameter was performed. Although the case study introduced a country-level problem which is a gap in the literature, some ideas were shared on how the proposed method can be used in case of an urban planning problem analogously. Further extensions of the technique were also investigated, e.g., regarding the definition of demands or the $\alpha$ parameter, moreover, the potentials of involving road network data were revealed. The performance of the proposed method has also been compared to the alternative K-medoid and MILP methods; it overperforms alternatives either in the practical performance of the result or computational time.

In the future, one main research direction is to extend the problem. For example, it is interesting how an optimum can be found under dynamic conditions. Demands, e.g., can change over time considering the amount or composition of the waste. If there is a prediction about it, that should be involved into the method to find a solution that is robust for the future as well. This is essential when resources cannot be easily moved (e.g., facilities). Besides, the method can support problems where the elimination of resources is also needed. It could be used analogously, e.g., to say where to close facilities in case of a decreasing demand. Moreover, in the presented container location case study (and other similar problems), it can also be important how the waste is transported from the containers to the targets, e.g., recycling facilities. In this work, only the side of the residents was considered and availability maximized; if the side of waste management, i.e., transportation cost of collection from containers is also wanted to be optimized, it forms a trade-off with availability. In this case, an optimization method for two levels could be developed. Last but not least, after distributing the resources among municipalities, their optimal location can be determined on the urban level as well.

## Supplementary Information

Supplementary Information 1.

Supplementary Information 2.

Supplementary Information 3.

Supplementary Information 4.

## Data Availability

The datasets analyzed during the current study and the Python implementation of the proposed algorithm made by Éva Kenyeres (Veszprém, Hungary) are available at https://github.com/kenyevica/Subtractive-clustering_Textile-waste-containers (accessed on 5 March 2026).

## References

[1] Zhang, D., Wang, M., Mango, J., Li, X. & Xu, X. A survey on applications of reinforcement learning in spatial resource allocation. *Comput. Urban Sci.* **4**, 14 (2024).

[2] Li, X., Walch, A., Yilmaz, S., Patel, M. & Chambers, J. Optimal spatial resource allocation in networks: Application to district heating and cooling. *Comput. Ind. Eng.* **171**, 108448. 10.1016/j.cie.2022.108448 (2022).

[3] Stopka, O., L’upták, V., Poliak, M. & Stopková, M. Optimization of collection routes using the centre of gravity method: A case study. *Sci. J. Silesian Univ. Technol. Ser. Transp.* **121**, 241–256. 10.20858/SJSUTST.2023.121.15 (2023).

[4] Wang, Y. et al. A review of regional and global scale land use/land cover (lulc) mapping products generated from satellite remote sensing. *ISPRS J. Photogramm. Remote Sens.* **206**, 311–334 (2023).

[5] Pan, R. et al. Mixed chlorine/chloramines in disinfected water and drinking water distribution systems (dwdss): A critical review. *Water Res.* **247**, 120736 (2023). 39491998 10.1016/j.watres.2023.120736

[6] Jeyaraj, R., Balasubramaniam, A., Ma, A. K., Guizani, N. & Paul, A. Resource management in cloud and cloud-influenced technologies for internet of things applications. *ACM Comput. Surv.* **55**, 1–37 (2023).

[7] Mondal, M., Sano, K., Kato, T. & Puppateravanit, C. Optimization of taxi allocation for minimizing co2 emissions based on heuristics algorithms. *Smart Cities* **6**, 1589–1611 (2023).

[8] Yadav, V., Karmakar, S., Dikshit, A. K. & Bhurjee, A. K. Interval-valued facility location model: An appraisal of municipal solid waste management system. *J. Clean. Prod.* **171**, 250–263. 10.1016/j.jclepro.2017.09.233 (2018). 10.1016/j.scitotenv.2017.02.207 28395953

[9] Lin, Z., Xie, Q., Feng, Y., Zhang, P. & Yao, P. Towards a robust facility location model for construction and demolition waste transfer stations under uncertain environment: The case of Chongqing. *Waste Manag.* **105**, 73–83. 10.1016/j.wasman.2020.01.037 (2020). 32032854 10.1016/j.wasman.2020.01.037

[10] Habib, M. S. et al. Large-scale disaster waste management under uncertain environment. *J. Clean. Prod.* **212**, 200–222. 10.1016/j.jclepro.2018.11.154 (2019).

[11] Ortega, F. A., Lega, M. & Itoh, H. *Waste management and the environment IX* (WIT Press ; Computational Mechanics International Inc., 2019).

[12] Moreno, J. C. et al. Optimizing uco container placement in urban environments: A genetic algorithm approach. *Lect. Notes Comput. Sci. (including subseries Lect. Notes Artif. Intell. Lect. Notes Bioinformatics)* **15347 LNCS**, 208–219, 10.1007/978-3-031-77738-7_18 (2025).

[13] Liu, L., Fong, S. & Ip, A. Grid based hierarchical clustering for spatial resource allocation. In *Proc. of the IADIS International Conference on E-Society, Avila, Spain* (2011).

[14] Malczewski, J. & Jackson, M. Multicriteria spatial allocation of educational resources: An overview. *Socioecon. Plann. Sci.* **34**, 219–235 (2000).

[15] Barrena, E., Canca, D., Ortega, F. A. & Piedra-De-La-Cuadra, R. Optimizing container location for selective collection of urban solid waste. *WIT Trans. Ecol. Environ.* **231**, 1–9. 10.2495/WM180011 (2019).

[16] Wang, Y., Liu, Y. & Pei, H. Designing a new robust solid waste recycling network under uncertainty: A case study about circular economy transition. *Socioecon. Plann. Sci.* **96**, 102066. 10.1016/j.seps.2024.102066 (2024).

[17] Liang, L., Wang, W., Jia, Y. & Fu, S. A cluster-based energy-efficient resource management scheme for ultra-dense networks. *IEEE Access* **4**, 6823–6832 (2016).

[18] Chambers, J., Zuberi, S., Jibran, M., Narula, K. & Patel, M. K. Spatiotemporal analysis of industrial excess heat supply for district heat networks in Switzerland. *Energy* **192**, 116705 (2020).

[19] Viktorin, A., Hrabec, D., Nevrlý, V., Šomplák, R. & Šenkeřík, R. Hierarchical clustering-based algorithms for optimal waste collection point locations in large-scale problems: A framework development and case study. *Comput. Ind. Eng.* **178**, 109142. 10.1016/J.CIE.2023.109142 (2023).

[20] Chiu, S. L. Fuzzy model identification based on cluster estimation. *J. Intell. Fuzzy Syst.* **2**, 267–278. 10.3233/IFS-1994-2306 (1994).

[21] Chen, Z., Zhu, Y., Di, Y. & Feng, S. Self-adaptive prediction of cloud resource demands using ensemble model and subtractive-fuzzy clustering based fuzzy neural network. *Comput. Intell. Neurosci.* **2015**, 919805. 10.1155/2015/919805 (2015). 25691896 10.1155/2015/919805 PMC4321097

[22] Vidovic, M., Simic, V., Dimitrijevic, B. & Ratkovic, B. Locating collection and sorting points for textiles waste. In *INTERNATIONAL SCIENTIFIC CONFERENCE “Innovative solutions for sustainable development of textiles industry”* (2009).

[23] Blazquez, C. & Paredes-Belmar, G. Network design of a household waste collection system: A case study of the commune of Renca in Santiago, Chile. *Waste Manag.* **116**, 179–189. 10.1016/j.wasman.2020.07.027 (2020). 32805553 10.1016/j.wasman.2020.07.027

[24] Feldman, O., Forero-Martinez, J. & Coombe, D. Alternative gravity modelling approaches for trip matrix synthesis. In *European Transport Conference 2012Association for European Transport (AET) Transportation Research Board* (2012).

[25] Yager, R. R. & Filev, D. P. Approximate clustering via the mountain method. *IEEE Trans. Syst. Man Cybern.* **24**, 1279–1284. 10.1109/21.299710 (1994).

[26] Costa, L. d. F. Further generalizations of the jaccard index. arXiv:2110.09619 (2021).

[27] Vaserstein, L. N. Markov processes over denumerable products of spaces, describing large systems of automata. *Probl. Peredachi Inf.* **5** (1969).

[28] Villani, C. The Wasserstein distances. In *Optimal Transport* 93–111 (Springer, Berlin Heidelberg, Berlin, Heidelberg, 2009).

[29] Korhonen, J., Honkasalo, A. & Seppälä, J. Circular economy: The concept and its limitations. *Ecol. Econ.* **143**, 37–46. 10.1016/J.ECOLECON.2017.06.041 (2018).

[30] Commission, E. Committee and the committee of the regions eu strategy for sustainable and circular textiles (2022).

[31] Boeing, G. Modeling and analyzing urban networks and amenities with osmnx. *Geogr. Anal.*10.1111/gean.70009 (2025).

[32] Moreno, C., Allam, Z., Chabaud, D., Gall, C. & Pratlong, F. Introducing the “15-minute city’’: Sustainability, resilience and place identity in future post-pandemic cities. *Smart cities* **4**, 93–111 (2021).

[33] Yang, K., Wang, R., He, H., Yang, X. & Zhang, G. Multi-supply multi-capacitated p-median location optimization via a hybrid bi-level intelligent algorithm. *Comput. Ind. Eng.* **160**, 107584. 10.1016/j.cie.2021.107584 (2021).

