# Supplementary material for: Subtractive clustering for spatial resource allocation problems in waste management
Source: Sci Rep. 2026 Mar 25;16:14986. doi: 10.1038/s41598-026-45718-4 (PMC13171916; doi:10.1038/s41598-026-45718-4)
Supplement: Supplementary file 4 — Supplementary Information 4. [file 41598_2026_45718_MOESM4_ESM.docx]

Supplementary Information

Title: Subtractive clustering for spatial resource allocation problems in waste management

Authors: Éva Kenyeres ^a^*, Alex Kummer^a^, János Abonyi^a^*

^a^ HUN-REN-PE Complex Systems Monitoring Research Group, University of Pannonia, Egyetem u. 10, P.O. BOX 158, Veszprém, H-8200, Hungary

*Corresponding authors: Éva Kenyeres ([kenyeres.eva@mk.uni-pannon.hu](mailto:kenyeres.eva@mk.uni-pannon.hu)), János Abonyi ([janos@abonyilab.com](mailto:janos@abonyilab.com))

**Result files**

Resulted datasets related to Figure 5, 6 and 7 are provided:

- *41598_2026_45718_MOESM3_ESM.xlsx* contains the list of predicted cities where containers should be placed when C is used as a normative control (**Figure 5**)
- *41598_2026_45718_MOESM2_ESM.xlsx* contains the list of predicted cities where containers should be placed when C is a fixed value (**Figure 6**)
- *41598_2026_45718_MOESM1_ESM.*xlsx contains the list of predicted cities for the first 1000 containers under various α values (**Figure 7**)

Underlying data files and created Python codes are available at <https://github.com/kenyevica/Subtractive-clustering_Textile-waste-containers> .
